# Expression of the *pstS *gene of *Streptomyces lividans *is regulated by the carbon source and is partially independent of the PhoP regulator

**DOI:** 10.1186/1471-2180-8-201

**Published:** 2008-11-19

**Authors:** Ana Esteban, Margarita Díaz, Ana Yepes, Ramón I Santamaría

**Affiliations:** 1 Instituto de Microbiología Bioquímica/Departamento de Microbiología y Genética, Consejo Superior de Investigaciones Científicas (CSIC)/Universidad de Salamanca, Edificio Departamental, Campus Miguel de Unamuno, 37007 Salamanca, Spain

## Abstract

**Background:**

PstS is a phosphate-binding lipoprotein that is part of the high-affinity phosphate transport system. *Streptomyces lividans *accumulates high amounts of the PstS protein in the supernatant of liquid cultures grown in the presence of different carbon sources, such as fructose or mannose, but not in the presence of glucose or in basal complex medium.

**Results:**

Functionality experiments revealed that this extracellular PstS protein does not have the capacity to capture phosphate and transfer it to the cell. Regulation of the *pstS *promoter was studied with Northern blot experiments, and protein levels were detected by Western blot analysis. We observed that the *pstS *gene was expressed in cultures containing glucose or fructose, but not in complex basal medium. Northern blot analyses revealed that the *pst *operon (*pstSCAB*) was transcribed as a whole, although higher transcript levels of *pstS *relative to those of the other genes of the operon (*pstC, pstA *and *pstB*) were observed. Deletion of the -329/-144 fragment of the *pstS *promoter, including eight degenerated repeats of a sequence of 12 nucleotides, resulted in a two-fold increase in the expression of this promoter, suggesting a regulatory role for this region. Additionally, deletion of the fragment corresponding to the Pho boxes recognized by the PhoP regulator (from nucleotide -141 to -113) resulted in constitutive *pstS *expression that was independent of this regulator. Thus, the PhoP-independent expression of the *pstS *gene makes this system different from all those studied previously.

**Conclusion:**

1.- In *S. lividans*, only the PstS protein bound to the cell has the capacity to bind phosphate and transfer it there, whereas the PstS form accumulated in the supernatant lacks this capacity. 2.- The stretch of eight degenerated repeats present in the *pstS *promoter may act as a binding site for a repressor. 3.- There is a basal expression of the *pstS *gene that is not controlled by the main regulator: PhoP.

## Background

Organisms detect and respond to extracellular nutritional conditions in different ways. *Streptomyces *spp. are some of the most abundant organisms in nature and have developed several mechanisms to survive under conditions of nutrient limitation, such as induction of the production of hydrolytic enzymes to degrade complex animal and plant debris, and antibiotic secretion to kill the closest organisms for their use as a new source of nutrients [1]. One of the most general and ubiquitous responses to nutrient limitation is mediated by the nucleotide guanosine 5'-diphosphate 3'-diphosphate (ppGpp), which triggers the onset of antibiotic production and morphological differentiation [2,3]. Another important signal involved in antibiotic production, and in general in secondary metabolism, is the level of phosphate present in the medium [4]. The production of a broad variety of metabolites responds to low levels of phosphate, a response that is mediated by the two-component system PhoR-PhoP [5]. One of the operons under the control of this system is the *pst *operon, which constitutes the high-affinity phosphate transport system induced under phosphate starvation [5-7]. The PstS protein is encoded by the first gene of the *pst *operon (*pstSCAB*) and constitutes the high-affinity phosphate-binding protein. In other organisms, a high expression of the PstS protein occurs under stress conditions, including alkali-acid conditions, the addition of subinhibitory concentrations of penicillin, and the response of pathogenic bacteria to the eukaryotic intracellular environment [8-11]. All these observations suggest that PstS would be one of the multi-emergency proteins that help cells to adapt to growth in different habitats.

In our previous work with *S. lividans *and *S. coelicolor*, we have described the extracellular accumulation of the high-affinity phosphate-binding protein PstS when the microorganisms are grown in the presence of high concentrations of certain carbon sources, such as fructose, galactose or mannose, although not with glucose. This accumulation is strikingly increased in a *S. lividans *polyphosphate kinase null mutant (*Δppk*). However, deletion of *phoP*, which encodes the response regulator of the PhoR-PhoP two-component regulatory system that controls the *pho *regulon, impairs its expression [6]. These observations clearly point to a phosphate-driven regulation of this system. Moreover, Sola-Landa *et al*. identified the so-called PHO boxes in the *pstS *promoter, and demonstrated that they are the binding sites for the phosphorylated form of PhoP [7,12].

Here we show that the PstS protein accumulated in the supernatant of *S. lividans *does not participate in the uptake of extracellular phosphate, and that only the PstS protein present in the cell is responsible for this process. We demonstrate that the *pstS *gene is also expressed in the presence of glucose but that the accumulation of RNA and protein is higher in the presence of fructose than in that of glucose in old cultures. Finally, using a multicopy *pstS *promoter-driven xylanase gene as a reporter we describe a functional study of this promoter aimed at elucidating the relevant regulatory regions by the carbon source and by PhoP.

## Results

### The extracellular PstS protein is not functional

**Figure 1:**
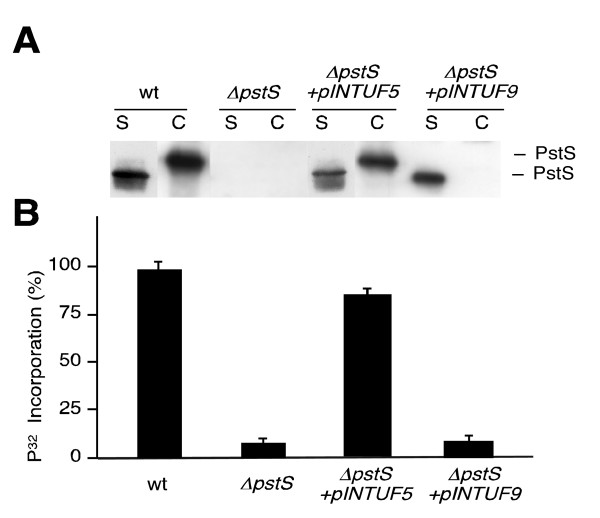
**Extracellular PstS does not participate in phosphate incorporation**. A) Western blot to detect extracellular (S) and cell-bound (C) PstS in the indicated *S. lividans *strains. (20 μg of total protein were loaded per lane). B) Uptake of ^32^P-labeled phosphate after 1 hour at 30°C. Strains assayed: wild-type *S. lividans *(wt); the *ΔpstS *deletion mutant (*ΔpstS*); the complemented transformant *ΔpstS *(*ΔpstS+*pINTUF5), and the same mutant containing the integrative fusion xylanase signal peptide-PstS (*ΔpstS+*pINTUF9). The results presented are the means of three independent experiments.

In principle, lipoproteins such as PstS are attached to the cell membranes, where they exert their function. However, our previous observations showed that the PstS protein was strongly accumulated in the supernatants of *S. lividans *cultures grown in the presence of certain carbon sources. We therefore decided to study whether this fraction of the protein also had the capacity to bind extracellular phosphate and transfer it to the cell. To address this issue, a construction expressing a [Xys1]-PstS fusion protein, which was completely secreted to the supernatant, was obtained (Methods). In this construction, the *pstS *promoter drives the expression of an in-frame fusion between the DNA fragment of the *xysA *gene encoding the signal peptide of the xylanase Xys1 from *S. halstedii *JM8 [13] and the region of the *pstS *gene that encodes the secreted form of the PstS protein. This fusion gene was cloned into a *Streptomyces *integrative plasmid to obtain plasmid pINTUF9 (Table 1), and this was introduced into the *S. lividans pstS *mutant (Table 2). As controls, *S. lividans *wild-type, the *pstS *mutant, and the *pstS *mutant transformed with plasmid pINTUF5 (Table 1), which produces the wild-type PstS protein, were used. The expression and location of the PstS protein were followed by Western blot analysis of the supernatants and cellular fractions of the different strains after 72 hours of culture. The original PstS protein was detected in the supernatants and in the cell extracts of the wild-type strain and in the *pstS *mutant transformed with pINTUF5. However, the PstS fusion protein, produced from pINTUF9 in the *pstS *mutant, was only detected in the culture supernatant (Fig. 1A). This result clearly demonstrates the capacity of the 45-amino acid signal peptide of the xylanase encoded by the *xysA *gene to secrete other proteins: in this case, PstS. The N-terminus of the secreted PstS protein obtained from the strain carrying pINTUF9 was identical to the wild-type PstS extracellular protein [6], except that it had two extra amino acids (A, G) at its N-terminus in order to keep the signal peptide processing site present in the original xylanase. Clearly, the size of the PstS protein observed in the cells and in the supernatant of the strains carrying the original *pstS *gene was different (Fig. 1A). This is due to the fact that the protein released to the supernatant does not have the first 41 amino acids [6].

**Table 1:** Plasmids

**Plasmid**	**Characteristics**	**Reference**
pKC796	Shuttle vector for *E. coli*/*Streptomyces *Apramycin resistance. Integrative plasmid in *Streptomyces*.	[29]

pKC796Hyg	Shuttle vector for *E. coli*/*Streptomyces *Hygromycin resistance. Integrative plasmid in *Streptomyces*.	[6]

pINTUF5	pKC796Hyg derivative containing the original *pstS *gene from *S. lividans*.	[6]

pINTUF2	pKC796 derivative containing the *pstS *promoter from *S. lividans *controlling the expression of the *xysA *xylanase gene from *S. halstedii*. [13]	This study

pINTUF9	pKC796Hyg derivative. The *pstS *promoter from *S. lividans *controls the expression of a fusion gene that contains the region of the *xysA *xylanase gene that encodes the signal peptide (45 amino acids) and the region that encodes the PstS secreted to the supernatant (from amino acid 42 up to the end).	This study

pN702GEM3	Shuttle vector for *E. coli*/*Streptomyces *neomycin resistance. Multicopy plasmid	[30]

pNUF5	pN702GEM3 derivative. The *pstS *promoter from *S. lividans *controls the *xysA *xylanase gene.	[6]

pNUF7	pN702GEM3 derivative. It contains the complete *pstS *gene from *S. lividans*.	[6]

pNX30	pN702GEM3 derivative. The *xysA *xylanase ORF does not have any promoter. Used as a negative control.	This study

pNUF11	pNUF5 derivative. The 29 bp that include the two PHO boxes of the *S. lividans pstS *promoter have been deleted (deletion includes from-141 to -113).	This study

pNUF13	pNUF5 derivative. The distal 186 bp of the *pstS *promoter have been deleted (from -329 to -144, both included). This deletion eliminates the 8-times degenerated sequence with the consensus: -ACYCASCCMNSV-.	This study

The next step was to study whether the secreted PstS protein, generated from pINTUF9, had the ability to participate in the uptake of extracellular phosphate. To do so, P^32^-phosphate incorporation in these four strains grown in the presence of fructose was measured. As demonstrated previously, phosphate incorporation by the *pstS *mutant was very low (about 8%) in comparison with the wild-type [6]. Transformation of this mutant with the pINTUF5 plasmid restored phosphate incorporation to almost wild-type levels. However, the *pstS *mutant containing pINTUF9, which produces only the secreted PstS, had a similar level of phosphate incorporation to that obtained with the *pstS *mutant (Fig. 1B). The fact that the PstS protein produced by this plasmid was unable to complement the defect in phosphate incorporation of the *pstS *null mutation suggests that the secreted PstS did not have the ability to capture extracellular phosphate and transfer it to the cell.

### *pstS *is expressed in the presence of glucose or fructose but not in basal medium

Up to this point we had focused our studies on the different levels of accumulation of the PstS protein in the culture supernatants, but we had not yet studied the amount of this protein bound to the cell under different culture conditions in depth. Aware that only the PstS protein bound to the cell must be functional, we focused our attention on this fraction.

**Figure 2:**
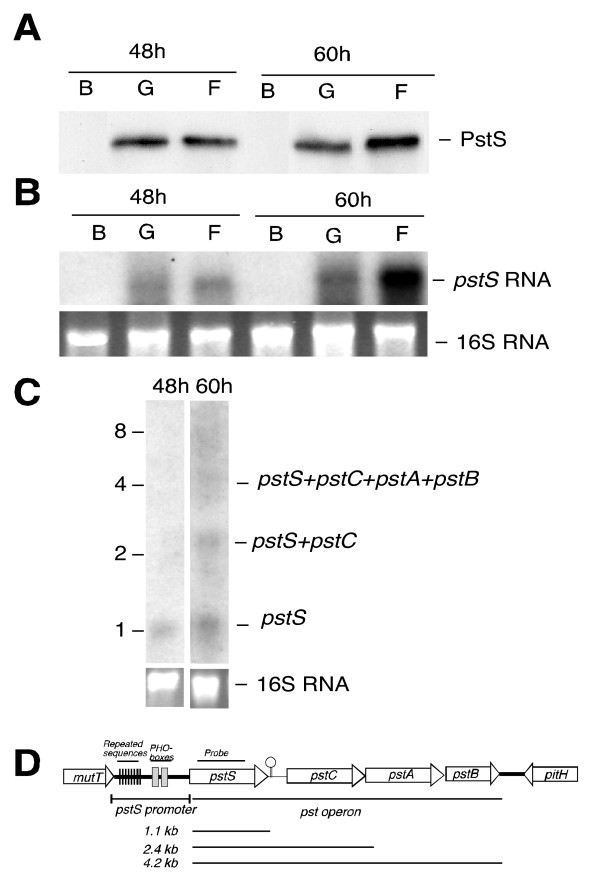
**Western and Northern analyses of PstS expression**. A) Western blot to detect cell-bound PstS in *S. lividans *TK24 grown under the indicated conditions (B, basal; G, basal + 5% glucose, F, basal + 5% fructose) and times (48, 60 hours). 20 μg of total protein were loaded per lane. B) Northern analysis of *pstS *expression in the above cultures. 16S RNA was used as a loading control (lower part). C) Transcriptional analysis of the *pstS *operon genes in the presence of 5% fructose at the indicated times (48, 60 hours). The bands detected are indicated at the right. 16S RNA was used as a loading control (lower part). D) Schematic representation of the *pst *operon and surrounding genes. A putative transcriptional terminator is proposed between *pstS *and *pstC*. The sizes of the RNA bands obtained in the Northern are indicated.

The first approach was to immunodetect PstS in wild-type *S. lividans *cells grown under different conditions. Cultures were performed in basal complex medium (YE) and in this medium supplemented with glucose or fructose, and samples were taken every 24 hours. No PstS protein was detected in cells obtained at 24 hours under any of the conditions used (data not shown). At 48 hours of culture, the protein was absent in cells from the basal medium but was clearly present in cells grown in the presence of glucose or fructose. At this time, the intensity of the PstS band was similar in the presence of both carbon sources (Fig. 2A). Later, at 60 hours, the PstS protein was more abundant in the presence of fructose than in that of glucose and was still absent in cells from basal YE medium (Fig. 2A). The same result was obtained for cultures of 72 and 96 hours (data not shown). As suggested previously, a possible explanation for this induction could be a difference in the rate of phosphate consumption in the presence of the different carbon sources [6]. Measurements of the residual phosphate levels of the three cultures after 60 hours showed that whereas 85 μM was detected in YE, only 15 μM was detected when the cells were grown in the presence of glucose or fructose. Therefore, since the phosphate levels in cultures with both carbon sources (glucose and fructose) were similar we suggest the existence of other regulators that could account for the higher amount of PstS detected in the presence of fructose in 60-h cultures and older, although the possibility of an effect of residual phosphate on PstS expression cannot be completely ruled out.

The effect of the carbon source was also studied at transcriptional level using Northern blot analysis. Total RNA was obtained from the same cultures at 24, 48 and 60 hours. Total RNA was separated in an agarose denaturing gel and hybridized with a ^32^P-labeled probe specific for *pstS *(see Materials and Methods). No hybridization bands were observed for any of the samples at 24 h (not shown). The transcription of *pstS *was clearly observed (as a band of about 1.1 kb) in 48-h and older cultures when supplemented with glucose or with fructose, but not in YE basal medium. The intensity of this 1.1 kb hybridization band, corresponding to the pstS transcript, was similar in the presence of glucose and in the presence of fructose at 48 h. However, at 60 h the band was much more intense in the presence of fructose than with glucose, indicating a higher induction of *pstS *transcription in the presence of this carbon source in old cultures (Fig. 2B).

At least three hybridization bands of 1.1, 2.4, and 4.2 kb were detected in 60 h-old cultures in the presence of glucose or fructose. The most prominent band was that of 1.1 kb, corresponding to monocistronic *pstS *transcript, as indicated previously. The 2.4 kb band corresponded fairly well to the size of a *pstSC *transcript (theoretical size 2.28 kb), while the size of the 4.2 kb band corresponded to the full-length *pstSCAB *operon transcript (theoretical size: 4.18 kb) (Fig. 2C, 2D). These results clearly indicate that the complete operon was transcribed in the presence of both carbon sources.

### Deletion of a repeated sequence in the *pstS *promoter of *S. lividans *duplicates its activity

**Figure 3:**
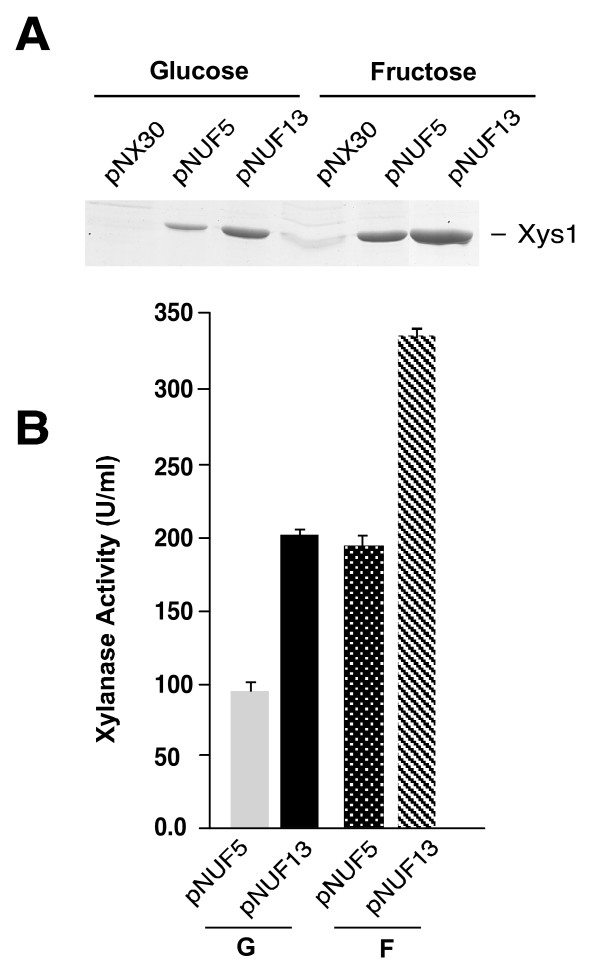
**Xylanase production under control of the *pstS *promoter**. A) Coomassie-Blue-R-stained SDS-PAGE showing the production of the Xys1 xylanase in supernatants of the *S. lividans *TK24 (wild-type) transformed with different plasmids: pNX30, the xylanase gene has no promoter; pNUF5, the xylanase gene is under the control of the full length *pstS *promoter; pNUF13, the xylanase is under the 186-bp-deleted *pstS *promoter (from -329 to -144). 5 μl of culture supernatant was loaded per track. B) Histogram showing the xylanase activity detected in the supernatant of the indicated strains. G, glucose; F, fructose. The results presented are means of three independent experiments.

We have previously proposed that the sequence ACTCACCCCCGC, repeated several times in the *S. coelicolor pstS *promoter and -with some discrepancies- up to eight times in the *pstS *promoter of *S. lividans*, might be involved in the carbon regulation of the expression of this promoter [6]. To study this in more detail, we deleted the portion of the *S. lividans pstS *promoter that contains the eight-times repeated degenerated sequence with the consensus sequence ACYCASCCMNSV. To do so, the -329/-144 region of the *pstS *promoter was deleted and the rest of the promoter was used to drive the expression of the ORF of the *xysA *xylanase gene [13] and used as reporter in a multicopy plasmid designated pNUF13 (Methods and Table 1). This plasmid (pNUF13), plasmid pNX30 (negative control: *xysA *without promoter), and plasmid pNUF5 (full-length *pstS *promoter controlling *xysA*) (Table 1) were introduced into *S. lividans *TK24 and cultures were grown in YE supplemented with 5% glucose or with 5% fructose in the presence of neomycin (20 μg.ml^-1^) for 72 h. The production of xylanase in the culture supernatants was studied by Coomassie blue-stained SDS-PAGE and by measuring the xylanase activity. The xylanase band obtained in the strain harbouring pNUF13 was significantly more intense than that obtained with pNUF5 in the presence of both carbon sources (Fig. 3A). Xylanase activity was quantified in all the supernatants, and we observed that no xylanase activity was detected in the cultures of the *S. lividans *TK24 strain transformed with pNX30 under both conditions (data not shown). However, xylanase activity was detected in the strain transformed with pNUF5 or with pNUF13 (Fig. 3B). Clearly, there was an increase in the xylanase activity detected in the strain carrying the *pstS *truncated promoter (pNUF13) under both culture conditions. This increase was more than two-fold when the strain was grown in the presence of glucose and 1.7-fold in the case of the cultures performed with fructose (Fig. 3B). In addition, we observed a higher expression in presence of fructose than in the presence of glucose for both truncated and complete pstS promoters. Thus, when the *S. lividans *TK24 strain was transformed with pNUF5, 2.15-fold more xylanase was produced with fructose than with glucose. When the plasmid used was pNUF13, the overproduction obtained with fructose was 1.7 fold, values of 340 U/ml of xylanase being attained. These results clearly indicate that the region containing the eight-times repeats may play an important role in controlling the level of expression of the *pstS *promoter in the presence of the different carbon sources.

### Basal expression of the *pstS *promoter is independent of the PhoP regulator

**Figure 4:**
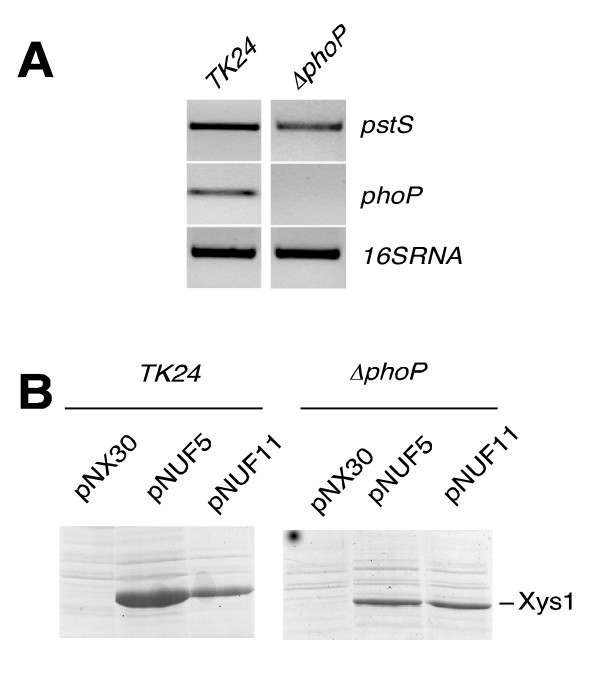
***pstS *expression in *S. lividans *TK24 (wt) and the *phoP *mutant**. A) Semiquantitative RT-PCR analysis of *pstS *and of *phoP *expression in the wild-type (TK24) and the *phoP *mutant (*ΔphoP*). RNAs from 60 h-cultures were used. The size of the amplified bands is 530 bp for *pstS*, 327 bp for *phoP*, and 416 bp for the 16S RNA. Forty amplification cycles were performed for the *phoP *gene in both strains and for the *pstS *gene in the *phoP *mutant. 20 amplification cycles were done for the rest of the RT-PCRs. B) Coomassie-Blue-R-stained SDS-PAGE of *S. lividans *TK24 and *ΔphoP *supernatants showing the production of the Xys1 xylanase under the control of the *pstS *promoter with the PHO boxes deleted (pNUF11). pNX30 and pNUF5 were used as controls (10 μl of 4-day-old culture supernatants were loaded per lane).

In order to study whether *pstS *expression was completely dependent on PhoP in *S. lividans*, we obtained total RNAs from the wild-type strain and from a *phoP *mutant cultured for 60 hours in the presence of fructose. RT-PCR for *pstS *was performed and one amplification band was observed for this gene in both strains. However, while the *pstS*-amplified band was clearly detectable after 20 cycles in the wild-type strain, 40 amplification cycles were necessary for it to be detected in the *phoP *mutant. As a control, we carried out RT-PCR for the *phoP *itself, observing that the amplification band was clearly obtained in the wild -type, although, as expected, no amplification band was obtained in the mutant (Fig. 4A). This observation demonstrates that residual *pstS *expression independent of PhoP- occurs, at least in *S. lividans*.

An alternative strategy to study this expression was to transform *S. lividans *TK24 and the *phoP *mutant with the plasmids pNX30 (negative control: *xysA *without promoter) and pNUF5 (full-length *pstS *promoter controlling *xysA*) to monitor xylanase production. No protein band corresponding to the xylanase was detected in either strain carrying the control plasmid pNX30. However, a protein band corresponding to the molecular weight of xylanase was readily detected in the parental TK24 strain transformed with plasmid pNUF5, and a weaker band -of the same size- was also observed in the *phoP *mutant transformed with the same plasmid (Fig. 4B). Western blot analyses with anti-xylanase antibodies confirmed that this band indeed corresponded to xylanase Xys1 (data not shown). Enzymatic activity assays confirmed that no xylanase activity was present in either strain transformed with pNX30. Values of 196 U/ml of xylanase were reached for the wild-type *S. lividans *transformed with pNUF5, and values of 19 U/ml for the *phoP *mutant transformed with the same plasmid. Thus, 10% of the activity of the *pstS *promoter must be out of the control of PhoP.

PhoP-independent basal expression was also demonstrated by generating a *S. lividans pstS*-modified promoter lacking the two PHO boxes described for the *S. coelicolor pstS *promoter [7]. To accomplish this, the -141/-113 region of the *S. lividans pstS *promoter containing the PHO boxes was deleted and this mutant promoter was used to monitor xylanase expression (Methods and Table 1). The plasmid generated, pNUF11, was introduced into both strains – wild-type *S. lividans *and the *phoP *mutant- and xylanase activity was analyzed in the culture supernatants. A protein band corresponding to the molecular weight of xylanase was observed in SDS-PAGE loaded with the supernatant of both strains transformed with pNUF11 (Fig. 4B). Quantification of the xylanase activity afforded identical values for both strains (19 U/ml). This result again indicates that although the activity of the *pstS *promoter is strongly reduced after deletion of the PHO-boxes it remains active, resulting in a basal level of PhoP-independent *pstS *transcription in *S. lividans*.

## Discussion

The normal localization of the PstS protein occurs through an extracellular association with the cell membrane by lipid anchorage and the protein participates in the uptake of extracellular phosphate, transferring this to the PstA and PstC transporters. An extracellular localization of part of the PstS protein has been described previously in other organisms such as *B. subtilis *[14], but such a high extracellular accumulation as that reported for old cultures of wild-type *S. lividans *and its *ppk *mutant have never been reported in other systems [6]. Recently, it has been found that *Pseudomonas aeruginosa *strains that display high virulence against intestinal epithelial cells accumulate extracellular PstS protein in appendage-like structures [15]. Those authors provided evidence that these appendages were involved in the adherence and disruption of the integrity of intestinal epithelial cells, and consequently in the pathogenicity of this bacterium. To date, no other studies of the functionality of extracellular PstS have been reported.

In the present study, we observed that the secreted PstS of *S. lividans *does not play any role in the uptake of extracellular phosphate. A possible explanation for this extracellular accumulation might be that the number of PstS molecules able to bind to the cell membrane might be limited. Consequently, the overproduction observed in the presence of several carbon sources could lead to a partial release of the protein to the supernatant as debris, this being resistant to proteolytic degradation owing to its configuration. To some extent, this hypothesis is corroborated by the fact that the level of PstS bound to the cells was similar in 60-h cultures and in cultures carried out over 6 days. However, the amount of protein found in the supernatant was much higher after six days (data not shown), suggesting that the promoter was still active and that the protein produced was being accumulated in the supernatant.

An interaction among different bacterial regulons involved in metabolism has been described in other organisms such as *B. subtilis*. In this organism, the phosphorus (*pho*) regulon is regulated by the carbon control protein-A (CcpA) through CcpA-responsive elements or *cre*. However, the mechanism of this control is not fully understood. Thus, whereas Choi et al. reported that CcpA controls the *phoPR *two-component system in a way independent of the *cre *sequence located in the *phoPR *promoter [16], Puri-Taneja et al. described that CcpA represses *phoP *transcription by binding directly to this *cre *sequence [17]. From our RNA experiments, it is clear that the *S. lividans pst *operon is transcriptionally induced by glucose or fructose in cultures that are close to or actually in the stationary phase. Under these conditions, the level of residual phosphate is lower than in basal medium, in which this operon is not induced, and this suggests that the phosphate level triggers the expression of the *pst *operon. However, while the residual phosphate of cultures with glucose and fructose were similar (in cultures of 60 hours and older) the expression of *pstS *was higher in the presence of fructose. Accordingly, although phosphate starvation seems to be the main signal for *pstS *expression, other regulators must also act in response to the carbon source present in the media. This was corroborated by deletion of the region that contains the eight-times repeated sequence in the *pstS *promoter proposed as the binding site of a carbon-responsive element [6]. This deletion resulted in a truncated *pstS *promoter with higher expression than the full-length promoter, suggesting that the sequence contained the binding site of a novel regulator. The identification of this putative regulator is currently under investigation at our laboratory.

To date, all the *pst *operons studied are members of the *pho *regulon. In some organisms such as *E. coli*, the number of PHO boxes in the promoter region has been related to inducibility by phosphate [18]. In *S. lividans *and *S. coelicolor*, the *pstS *promoter contains two 11-bp direct repeat units that constitute two PHO boxes in tandem, which are the binding sites for the phosphorylated PhoP protein [7]. As demonstrated here, deletion of these sequences in the *S. lividans pstS *promoter results in constitutive *pstS *expression that is similar in the wild-type and in the *phoP *mutant. From these results it may be concluded that although PhoP is the main regulator of this promoter, some basal expression of the *pstS *promoter (1/10 of the normal activity) escapes PhoP regulation. Similarly, it has been demonstrated that the *pst *operon of *Corynebacterium glutamicum *is partially induced in the *phoR *mutant (in this organism the regulator has been designated PhoR and the kinase as PhoS), suggesting that at least one other additional regulator must be involved in its expression [19]. These results differ from those reported recently for *S. coelicolor *by Sola-Landa et al. [12]. These authors demonstrated that the *S. coelicolor pstS *promoter, controlling a promoterless catechol dioxygenase gene *xylE*, was not expressed in a *S. coelicolor phoP *mutant. However, the *pstS *promoter from *S. lividans *is 28 bp longer than the corresponding promoter from *S. coelicolor *[6] and therefore different types of regulation cannot be ruled out. Our results also differ from those obtained from *Bacillus subtilis*, where the *pst *promoter has been used to control β-galactosidase expression [20]. Those authors reported that no expression was observed in a mutant strain lacking the *phoP *gene, although they did detect a low level of expression in the mutant of the *phoR *sensory kinase gene. Thus, it is possible that PhoP might be phosphorylated inefficiently by other sensory kinases in this strain [20].

Our Northern blot experiments demonstrated that the *S. lividans pst *operon is transcribed as a single 4.2 kb transcript that corresponds to the complete *pst *operon and that it may be processed at specific points, resulting in smaller RNAs; the most abundant one would be that corresponding to *pstS*. A similar expression of the *pst *operon has been described in *E. coli *and in *B. subtilis *[21,22]. Both organisms have palindromic sequences in the intergenic region between *pstS *and *pstC *that show 73% sequence identity and that can prevent ribonuclease activity and consequently stabilize *pstS *RNA upstream. In our study, the MFOLD program [23] predicted a strong stem-loop structure for the RNA corresponding to the *pstS-pstC *intergenic region, which possibly functions as a transcriptional terminator. This terminator would start 3 bp downstream from the UGA stop codon of *pstS*, ending at nucleotide 83 downstream from *pstS*, with a ΔG (change in Gibbs free energy) of -60.2 kcal/mol. Another explanation for our Northern results is that the transcription machinery might stop at the terminator between *pstS *and *pstC*, resulting in more *pstS *transcripts relative to the larger multicistronic *pst *transcripts. This is corroborated by the ratio of intensities of the different bands observed in the samples after 48 and 60 h. Thus, while the 1.1 kb band was detected at 48 hours, the larger 2.4 and 4.2 kb bands were detected at 60 h. of culture, and their intensities were lower than that of the former band (Fig. 2C).

## Conclusion

Although cultures of *S. lividans *accumulate high amounts of the PstS protein in the supernatant of cultures carried out with certain carbon sources such as fructose, the results of the present work demonstrates that the secreted form of this protein does not have the capacity to bind external phosphate and transfer it to the cells. Our study also reveals a novel regulatory system for *pstS *expression in *S. lividans*. In contrast to other systems described so far, where PhoP appears to be the only regulator, we detected a basal expression of *pstS*, which escapes the control of the main regulator PhoP. We have also identified a region of the *pstS *promoter containing eight degenerated repeats that may act as a binding site for as yet unknown repressor(s). In sum, our work reveals the complexity of the regulatory network in *Streptomyces *and uncovers a connection between phosphate and carbon regulation, which should be further investigated in the future. We are tempted to speculate that these novel regulators would contribute to the integration of the different nutritional signals that allow the organism to survive under adverse environmental conditions.

## Methods

### Bacterial strains and media

All strains used are listed in Table 2. *Streptomyce*s strains grown at 30°C on Solid Mannitol Soya Flour Agar medium (MSA), or R2YE [1] were used for normal cultures and sporulation. Submerged cultures were usually carried out in YE medium (1% yeast extract) supplemented with different amounts of the carbon source studied: normally fructose or glucose at 5%. Cells were grown at 28°C and 250 rpm in an orbital shaker (Adolf Kühner AG, Birsfelden, Switzerland) for as long as required for each assay (1–5 days) in baffled flasks with 1/10 volume of medium. *E. coli *was grown in Luria Broth (LB) or in Luria Agar at 37°C. Supplements of kanamycin (50 μg ml^-1^), neomycin (50 μg ml^-1^) or hygromycin (50–200 μg ml^-1^) were added when needed. Cell extracts were obtained in a Fast-Prep device (Q-Biogene).

**Table 2:** Bacterial strains.

**Strain**	**Genotype**	**Comments**	**Reference**
*S. lividans *66	SLP2^+ ^SLP3^+^ Wild type.	Parental strain of the *pstS *mutant.	[1]

*S. lividans ΔpstS*	SLP2^+ ^SLP3^+^ *ΔpstS*	Mutant defective in the high-affinity phosphate protein PstS.	[6]

*S. lividans *TK24	*str-6 *SLP2^- ^SLP3^-^ Wild type.	Parental strain of *phoP *mutant.	[1]

*S. lividans ΔphoP*	*str-6 *SLP2^- ^SLP3^-^*ΔphoP*	Mutant defective in the regulator of the two-component system PhoP/R.	[27]

*E. coli *DH5α	F^-^, ϕ 80 d *lac*ZΔM15, Δ(*lac*ZYA-argF)U169, *rec*A1, *end*A1, *hsd*R17(rk^-^, mk^+^), *sup*E44, λ-, *thi*-1, *gyr*A, *rel*A1	Used for cloning and plasmid isolation.	[28]

### DNA manipulations and transformations of *S. lividans *and *E. coli*

Total genomic DNA, plasmid isolation, transformation, and protoplast manipulation were performed as previously described [6]. The plasmids used are listed in Table 1.

### RNA isolation and Northern analysis

The RNA for the analysis of the *pst *operon was obtained from cultures of *S. lividans *TK24 grown in YE or in this medium supplemented with 5% glucose or 5% fructose at different times. The RNA was obtained at 24, 48 and 60 hours, according to the protocol provided with the RNeasy Protect bacteria Mini Kit (QIAGEN). RNase-free DNase (RQ1, Promega) was used to eliminate all DNA. The quality and quantity of RNA was analyzed in agarose gels and by spectrometry in an Agilent bioanalyzer.

Northern blot analysis of the *pstS *gene and of the complete operon was carried out with 4 μg of RNA denatured on 1% formaldehyde agarose gels and transferred to a Hybond-N membrane, essentially as described [24]. A *pstS *probe containing most of the open reading frame (918 bp, from nucleotide +189 to +1107) was obtained by PCR using the oligonucleotides MRG-30 and MRG-31 (Table 3) and plasmid pNUF7 (Table 1) as template, and was then labeled by random priming using the DNA Labeling Beads (dCTP) kit and [^32^P]dCTP (Amersham). Hybridization was carried out at 65°C in a solution containing 5× SCC (1× SCC in 0.15 M NaCl, pH 7, plus 0.015 M sodium citrate), 2× Denhardt's solution, 0.5% sodium dodecyl sulfate (SDS), and 0.1 mg/ml sheared salmon sperm DNA (AMBION). After hybridization, the blots were washed with: 5× SCC containing 0.1% SDS at room temperature for 20 min., 2× SCC containing 0.1% SDS at 42°C for 20 min., 0.2× SCC containing 0.1% SDS at 42°C for 20 min. and 0.1× SCC containing 0.1% SDS at 65°C for 20 min. The 0.24- to 9.5-kb RNA ladder (Life Technologies) was used for sizing the RNA in formaldehyde-agarose gels. The intensity of 16S RNA was used as a loading control.

**Table 3:** oligonucleotides used

Name	Sequence	Origin/use
MRG-27	TAATAACATATGGCGCTGAAGCTTCACTTGAGGGAG	Reverse oligonucleotide for cloning the *pstS *promoter. The sequence recognized by *Nde*I is underlined.

MRG-28	TTTTTAGATCTCAGCCCCGGGACCGGGCCCT	Forward oligonucleotide for cloning the *pstS *promoter. It was designed at the end of SCO4143 that is upstream from the *pstS *gene. The sequence recognized by *Bgl*II is underlined.

MRG-34	TTTTTCTAGATCAGCTCAGGCCCGAGATGGTC	Reverse oligonucleotide to clone the region of *pstS *gene that encodes the secreted PstS protein. It contains a *Xba*I site for cloning.

RS005	CCTTCGGCGCCTTCATCTCATC	Forward oligonucleotide of the *S. lividans pstS *promoter from nucleotide -112 to -91. Used in a PCR to delete the PHO boxes.

RS007	GATGAGATGAAGGCGCCGAAGGGGACGGTGCGGTGAGGTCAC	Reverse oligonucleotide to delete the PHO boxes in *pstS *promoter (from nucleotide -141 to -113). The oligonucleotide contains from nucleotide -161 to -142 and from -112 to -91.

RS008	ATCCCCCGGGAGCAACATCAAGTGCGACGACGCC	Forward oligonucleotide to clone the region of *pstS *gene that encodes the secreted PstS protein. It contains a *Sma*I site for cloning.

RS009	TCCCCCGGGCCACAGGGGTTCACCCGGCG	Forward oligonucleotide of the *S. lividans pstS *promoter from nucleotide -143 to -124. Used to delete the 186 bp region upstream from the PHO boxes in a PCR with Oli MRG-27. It contains a *Sma*I site for cloning.

AE007	GCCTGGGTCAAGCAGTACGTCG	Forward oligonucleotide of the *S. lividans pstS *gene from nucleotide +199 to +220. Used in RT-PCR analysis.

AE008	GATGGCGCCGGGGGTCTGCTT	Reverse oligonucleotide of *S. lividans pstS *gene from nucleotide +715 to +735. Used in RT-PCR analysis.

AE024	TCGTCGGGCTGGAGATAGGG	Forward of *S. lividans phoP *gene from nucleotide +254 to +273. Used in RT-PCR analysis.

AE025	CGTGGACGTCGAGGGTCTTG	Reverse oligonucleotide of *S. lividans phoP *gene from nucleotide +561 to +580. Used in RT-PCR analysis.

16S F	TCACGGAGAGTTTGATCCTGGCTC	Forward oligonucleotide of *S. lividans 16S *gene from nucleotide +20 to +44. Used in RT-PCR analysis.

16S R	CCCGAAGGCCGTCATCCCTCACGC	Reverse oligonucleotide of *S. lividans 16S *gene from nucleotide +436 to +460. Used in RT-PCR analysis.

MRG-30	GCCATCGACGCCTGGGTCAAG	Forward oligonucleotide of *S. lividans pstS *gene from nucleotide +189 to +210. Used to obtain pstS probe for Northern blot analysis.

MRG-31	CAGGCCCGAGATGGTCTCGCG	Reverse oligonucleotide of *S. lividans pstS *gene from nucleotide +1086 to +1107. Used to obtain pstS probe for Northern blot analysis.

### RT-PCR analysis

RT-PCR analyses were carried out using the Superscript™ One-Step RT-PCR with the Platinum^® ^Taq System (Invitrogen). RNA samples from *S. lividans *TK24 and its *phoP *mutant were collected at 48 and 60 h, as indicated previously. For each RT-PCR reaction, 200 ng of RNA were used in a final volume of 20 μl. The program used was as follows: 30 min cDNA synthesis at 55°C, followed by 2 min at 95°C and 20–40 cycles of: 45 s at 94°C (denaturation), 30 s at 65°C (annealing) and 40 s at 65°C (elongation). The reaction was completed by a 10-min incubation at 72°C. Two microlitres of each sample were visualized by electrophoresis in an ethidium bromide-stained 1.6% agarose gel in 1× TAE. The oligonucleotides used are listed in Table 3.

### Construction of an integrative plasmid with a xylanase signal peptide-*pstS *fusion

To perform PstS functional studies, the integrative plasmid pINTUF9 (Table 1) was constructed. In this plasmid, all the PstS protein produced was secreted to the supernatant owing to an in-frame fusion with the 45-amino acid signal peptide of the xylanase Xys1 from *S. halstedii *JM8 [13]. The plasmid was obtained after several cloning steps. Briefly, the region of the *pstS *gene that encodes the mature PstS protein located in the supernatant (from amino acid 42 up to the end) was isolated from plasmid pNUF7 [6] by PCR using the oligonucleotides RS-008 and MRG-34 (Table 3). The PCR band was digested with *Sma*I/*Xba*I and ligated with plasmid pINTUF2 (Table 1), digested with *Nae*I/*Xba*I (*Nae*I cuts just after the sequence that encodes the signal peptide of the xylanase and *Xba*I cuts at the end of the *xysA *gene). The new construct had the *pstS *promoter controlling a fusion gene that contained the signal peptide of the xylanase (45 aa) fused in-frame with the fragment of DNA that encodes the PstS protein released to the supernatant. This construction, flanked by terminators, was transferred to plasmid pKC796Hyg (Table 1) by digestion of both DNAs with *EcoR*V/*Kpn*I, ligation, and selection in *E. coli*. The plasmid obtained, pINTUF9, and the empty vector, pKC796Hyg, were introduced into *S. lividans ΔpstS *by protoplast transformation, and the integrated strains were selected for hygromycin resistance. These plasmids were integrated into the genome of the *S. lividans pstS *mutant at the ϕ C31 integration site.

### Analysis of the activity of the *pstS *promoter and of several deletions

The expression of the *pstS *promoter and several deletions (see below) was studied using the xylanase gene (*xysA*) from *S. halstedii *as a reporter [13]. The intergenic region (329 nucleotides) between the homologous genes to *S. coelicolor *SCO4142 (*pstS *gene) and SCO4143 (a possible mutT-like protein) was previously cloned from *S. lividans *to obtain the multicopy plasmid pNUF5. That region was called the full-length *pstS *promoter [6]. Deletion of the distal region (from -329 to -144), which contained a degenerated sequence of 12 nucleotides repeated 8 times (see results), was accomplished using the oligonucleotides RS009 (forward) and MRG-27 (reverse) (Table 3) and was used to replace the whole *pstS *promoter. The new plasmid obtained was designated pNUF13. Another plasmid, designated pNUF11, was obtained by deletion of the 29 bp (from -141 to -113, both included) that form the two PHO-boxes present in this promoter [7]. This internal deletion was constructed in a two-step PCR, using two of the different PCR products obtained: one with oligonucleotides MRG-28/RS007 and the other with RS005/MRG-27 (Table 3). Both were mixed (they have 21 bp overlapping ends) and used as templates in a second PCR with the external oligonucleotides MRG-28/MRG-27. In all cases, the amplified fragments were sequenced. In these experiments, *S. lividans *TK24 (wild-type strain) and the *S. lividans phoP *mutant were used as hosts. The xylanase activity produced by the different versions of the promoter was used as a reporter and was quantified from liquid cultures.

### Phosphate uptake

Phosphate uptake was measured in *S. lividans *cultures grown in liquid YE + 5% F medium for 72 h (30°C, 200 rpm). ^32^P-labeled Na_2_HPO_4 _(Amersham Biosciences) was added (2 × 10^5 ^cpm) and phosphate uptake was measured after 1 hour at 30°C. Cells were recovered by filtration through Whatman GF/C filters, washed twice with 0.9% NaCl, and the radioactivity from the filter was quantified on a liquid scintillation counter (Wallac 1409-001).

### Residual Phosphate determination

Phosphate concentrations in the culture media were determined by a modification of the malachite green/molybdate method, using KH_2_PO_4 _as standard [6]. The reactions were carried out by mixing the supernatant (up to 100 μl) with 800 μl of ammonium molybdate (4.2% in 4N HCl), malachite green (0.045% in ddH_2_O), Tween 20 (10% in ddH_2_O) solution and 100 μl of citrate solution (34% in ddH_2_O), and the mixture was further incubated at RT for 30 min. Absorbance was measured at 660 nm.

### Enzyme assays

Xylanolytic activity was determined in the culture broth by the dinitrosalicylic acid (DNS) method, using oat-spelt xylan (Sigma) as substrate and xylose as standard [25,26]. One enzymatic unit of xylanase was defined as the amount of enzyme required to release 1 μmol of reducing sugars (expressed as xylose equivalents) per minute. Activity was calculated in enzyme units per ml of supernatant of culture.

### Protein analysis

Electrophoresis in denaturing polyacrylamide gels (SDS-PAGE) was performed as described previously [13]. Western blot analyses were done on protein transferred to polyvinylidene difluoride membranes and probed with appropriate antibodies. Typically, anti xylanase (Xys1) antibodies [13] or anti-PstS antibodies were used. Anti-PstS antibodies were raised in rabbits against *S. lividans *PstS protein purified from the supernatants of cultures carried out in the presence of 5% fructose. Fast-performance liquid chromatography (FPLC) was used to purify the protein (data not shown). Typically, a 1/150.000 dilution was used for this antibody. Horseradish peroxidase-conjugated secondary donkey-anti-rabbit antibody was used. The blots were developed with ECL reagents (GE Healthcare), used according to the manufacturer's instructions.

Enzymes and reagents were purchased from Bio-Rad, Roche, GE Healthcare, Invitrogen, Merck, Panreac, Promega, Quiagen or Sigma, and were used following the manufacturer's guidelines.

## Authors' contributions

AE made most of the experimental work. AY made xylanase quantification. MD and RIS designed and made the plasmids with the deleted *pstS *promoter and wrote the manuscript. All authors have read and approved the final version of the manuscript.
